# Modified Venous Excess Ultrasound (mVExUS) in the Prediction of Ventilator Weaning Failure: a Cohort Study

**DOI:** 10.24908/pocusj.v11i01.19496

**Published:** 2026-04-22

**Authors:** Tiago Hermes Maeso Montes, Wagner Luis Nedel, Márcio Manozzo Boniatti

**Affiliations:** 1 Department of critical care, Hospital Nossa Senhora da Conceição, Porto Alegre, Brazil; 2 Department of critical care, Hospital de Clínicas de Porto Alegre, Porto Alegre, Brazil; 3 Universidade La Salle, Canoas, Brazil

**Keywords:** VExUS, POCUS, Lung ultrasound, Weaning, Mechanical ventilation, Critically ill

## Abstract

**Background:**

Ventilator weaning failure is a significant challenge in critically ill patients, and venous congestion has emerged as a potentially modifiable factor influencing weaning outcomes. This study aimed to investigate the association between venous congestion identified by the modified venous excess ultrasound (mVExUS) score and ventilator weaning failure in critically ill patients.

**Methods:**

We prospectively enrolled patients aged 18 years or older who had received mechanical ventilation (MV) for at least 48 hours. A mVExUS score, excluding the intrarenal component, and a lung point of care ultrasound (POCUS) assessment were performed before a spontaneous breathing trial (SBT). The primary outcome was weaning failure, defined as a failed SBT or the need for MV (invasive or non-invasive) within 72 hours.

**Results:**

Among 111 patients, 57 (51.4%) experienced ventilator weaning failure. Ventilator weaning failure occurred in 63.9% of patients with mVExUS scores of 2–3, compared to 45.3% with scores of 0–1 (p = 0.067). In adjusted analyses, mVExUS scores of 2–3 were independently associated with weaning failure (OR 2.756, p = 0.03). A post-hoc analysis combining mVExUS and lung POCUS scores showed a weaning failure incidence of 76.5% in patients with mVExUS scores of 2–3 and lung POCUS scores ≥7, compared to 34.1% in patients with mVExUS scores of 0–1 and lung POCUS scores <7 (p = 0.002).

**Conclusion:**

mVExUS scores of 2–3 are significantly associated with a higher risk of weaning failure and post-extubation respiratory failure. Combining VExUS and lung POCUS scores may enhance the assessment of weaning readiness in critically ill patients.

## Introduction

The liberation of patients from mechanical ventilation (MV) remains a significant challenge in the management of critically ill individuals. Failure to successfully wean from MV is associated with adverse clinical outcomes [1,2]. Multiple mechanisms contribute to extubation failure, among which an inadequate cardiovascular response plays a central role [3–5]. During the ventilator weaning process, the withdrawal of positive pressure ventilation functions as a cardiovascular stress test, and can trigger a weaning-induced pulmonary edema (WIPO) [6]. Recently, Shi et al. reported that WIPO accounted for over one-third of weaning failure cases, particularly among patients with pre-existing heart disease or chronic obstructive pulmonary disease [7]. Consequently, identifying patients at risk for cardiovascular dysfunction during MV weaning using a cost-effective, non-invasive, and widely accessible method is of great clinical importance.

Lung point of care ultrasound (POCUS) has emerged as a valuable tool for detecting WIPO. Several studies have demonstrated that higher lung POCUS scores are associated with ventilator weaning failure [8–10]. While lung POCUS is well established in assessing pulmonary congestion during the weaning process, its ability to evaluate systemic venous congestion is limited. In contrast, the venous excess ultrasound (VExUS) score provides a more comprehensive assessment of systemic congestion by integrating measurements of inferior vena cava (IVC) diameter with pulsed-wave Doppler patterns of the hepatic, portal, and intrarenal veins [11]. A modified version of the VExUS score (mVExUS), which excludes the intrarenal component, offers a simplified alternative while maintaining good diagnostic performance in identifying venous congestion. This integrated approach allows the VExUS score to quantify systemic congestion and to reflect the severity of underlying cardiovascular dysfunction [12,13]. Given that patients with systemic venous congestion are at increased risk of developing WIPO, the VExUS score may provide complementary insights to lung POCUS [14,15]. This can enhance the assessment of patients during the ventilator weaning process.

Therefore, this study aimed to investigate the association between venous congestion identified by the mVExUS score and ventilator weaning failure in critically ill patients undergoing a spontaneous breathing trial (SBT), independent of the lung POCUS score.

## Methods

We conducted a prospective cohort study in the intensive care unit (ICU) of a tertiary hospital in Porto Alegre, Brazil, from July 5, 2022 to July 13, 2023. The study was conducted in accordance with the principles of the Declaration of Helsinki. The research protocol was approved by the institutional ethics committee, and written informed consent was obtained from each participant or their legally authorized representative.

We prospectively enrolled patients aged 18 years or older who received MV for at least 48 hours and were deemed ready for a SBT. Exclusion criteria included liver cirrhosis, portal vein thrombosis, presence of a tracheostomy, or a do-not-reintubate order. Patients with a do-not-reintubate order would not be eligible for reintubation and therefore could not meet one of the components of the primary outcome. The decision to initiate the SBT was made by the attending physician based on routine clinical judgment. All eligible patients were systematically screened, but because a single investigator performed all ultrasound assessments, patients were only enrolled on days when the investigator was avaliable. Therefore, a convenience sampling strategy was applied.

Before the initiation of the SBT, both mVExUS and lung POCUS assessments were performed by one of the investigators (THMM), a clinician with extensive experience in critical care ultrasonography who was not involved in patient management. All ultrasound images were subsequently reviewed by a senior ICU physician (MMB), also highly experienced in critical care ultrasonography and blinded to clinical outcomes, to ensure data quality and objectivity.

mVExUS assessments were performed using a portable ultrasound machine (M-Turbo, Sonosite, Seattle, USA) equipped with a 2–5 MHz curved-array transducer. Patients were positioned in the dorsal decubitus position with the head of the bed elevated to 30 degrees. The diameter of the IVC was initially measured in its intrahepatic segment, approximately 2 cm from the junction with the hepatic veins, using both short- and long-axis views from a subxiphoid window. If the subxiphoid view was inadequate, the probe was repositioned laterally on the right side of the abdomen over the liver to obtain a suitable image. The maximal IVC diameter during the respiratory cycle was recorded.

If the IVC was nonplethoric (<2 cm), the patient was classified as mVExUS 0. If the IVC was plethoric (diameter ≥2 cm), further Doppler evaluations of the hepatic vein and portal vein were performed, preferably from the mid- to posterior axillary line. Electrocardiogram (ECG) monitoring was used concurrently to aid in waveform interpretation, and all images were analyzed offline.

Hepatic vein Doppler was obtained using pulsed-wave (PW) Doppler to identify A, S, and D waves. The patterns were classified as follows [11]: Normal: S >D; Mild abnormality: S <D; Severe abnormality: reversed S wave. Portal vein Doppler was also assessed using PW Doppler. Peak (V_max_) and nadir (V_min_) velocities during the cardiac cycle were recorded, and the pulsatility fraction was calculated using the formula: Pulsatility fraction = (V_max_ – V_min_) / V_max_. Interpretation followed the criteria proposed by Beaubien-Souligny et al. [11]: Normal: <0.3; Mild abnormality: 0.3 to <0.5; Severe abnormality: ≥0.5.

We did not include the intrarenal vein Doppler (IRVD) in our mVExUS protocol. This decision was intentional as we aimed to simplify the examination for routine bedside application in critically ill patients. Previous studies have shown that the exclusion of IRVD does not substantially compromise the score's ability to detect clinically significant systemic congestion [16–18].

mVExUS was applied, based on the VexUS; a scheme originally described by Beaubien-Souligny et al. and used by Bhardwaj et al. [11,16,18]. This simplified classification excludes the IRVD component and defines mVExUS 0 as IVC <2 cm; mVExUS 1 as IVC ≥2 cm with normal Doppler waveforms; mVExUS 2 as IVC ≥2 cm with at least one mild Doppler abnormality (in the hepatic or portal vein); and mVExUS 3 as IVC ≥2 cm with at least one severe Doppler abnormality. The decision to adopt this simplified protocol was made to facilitate bedside assessment and align with prior studies using similar approaches.

Lung POCUS was performed using the same curved-array probe. A comprehensive scan was performed in eight zones—four for each hemithorax (superior and inferior regions in both the anterior and lateral areas) using the anterior axillary line as the landmark. A scoring system based on aeration patterns was applied as follows: A-lines (0 points), separated B-lines (1 point), coalescent B-lines (2 points), and lung consolidation (3 points) [10]. The total lung POCUS score ranged from 0 (normal aeration) to 24 (most severe aeration loss) [19,20].

The SBT was performed using either a T-piece connected to an oxygen source or low-level pressure support (8 cm H_2_O) with positive end-expiratory pressure (PEEP) ≤5 cm H_2_O and FiO_2_ ≤40%. SBT failure was defined by the occurrence of any of the following criteria: agitation; decreased level of consciousness (Glasgow Coma Scale <13); respiratory rate >35 breaths/min or use of accessory muscles; oxygen saturation <90%; heart rate >140 beats/min or a >20% increase from baseline; systolic blood pressure <90 mmHg; or onset of arrhythmias. Patients who failed the SBT were reconnected to MV. Those who successfully completed the SBT were extubated at the discretion of the attending physician, independent of the investigators. Prophylactic non-invasive ventilation (NIV) was defined as its immediate initiation following extubation, maintained for 4 hours, and used preemptively to reduce respiratory distress.

The primary outcome was ventilation weaning failure, defined as either SBT failure or the need for MV (invasive or non-invasive) within 72 hours following extubation. The secondary outcome was post-extubation respiratory failure, defined by the presence of at least two of the following criteria: respiratory acidosis; oxygen saturation <90% with FiO_2_ ≥50%; respiratory rate >35 breaths/min for two consecutive hours; or clinical signs of respiratory fatigue.

### Statistical analysis

Categorical variables were presented as absolute and relative frequencies, while continuous variables were summarized using means and standard deviations or medians and interquartile ranges, as appropriate. The distribution of continuous variables was assessed using the Kolmogorov-Smirnov test. Comparisons of continuous variables were performed using either Student's *t*-test or the Mann-Whitney *U* test, depending on the distribution. Categorical variables were compared using the χ^2^ test when the expected frequency in each cell was ≥5, and Fisher's exact test was used when this assumption was not met. There were no missing data for any of the variables included in the analysis.

A multivariate binary logistic regression model was constructed to identify variables independently associated with the primary and secondary outcomes, both of which were binary. The mVExUS score was retained as a variable of interest in the multivariable analysis. Additional covariates were defined *a priori* based on their plausible association with the primary outcome: SAPS 3, duration of MV, lung POCUS score, and cumulative fluid balance prior to the SBT. Linearity of continuous variables with the logit was tested using the Box-Tidwell procedure, and multicollinearity was assessed using variance inflation factors.

Based on the study by Ferré et al., a ventilator weaning failure rate of 45% was assumed for the congestive group [9]. For the non-congestive group, we estimated a failure rate of 15%, reflecting clinical expectations in less congested patients. Assuming a 2:1 ratio between non-congestive and congestive patients, with a two-sided alpha of 0.05 and 80% power, the required sample size was estimated at 55 patients without congestion and 28 with congestion, totaling 83 patients. After adding 20% to account for potential losses or missing data, the final required sample size was approximately 100 patients. Although this calculation provided a minimum target, all eligible patients assessed during the predefined study period were enrolled whenever the investigator was available.

The mVExUS grade was categorized into two groups: 0–1 (no or mild congestion) and 2–3 (moderate or severe congestion). Similarly, the lung POCUS score was dichotomized. The optimal cutoff point for the lung POCUS score in predicting the primary outcome was determined using the Youden index. Statistical significance was set at *p* <0.05, and all analyses were conducted using SPSS software, version 20.0.

## Results

During the study period, 116 patients underwent a SBT. Five were excluded due to inadequate image quality for score determination, resulting in a final cohort of 111 patients. Their clinical characteristics are presented in Table 1. Among them, 57 patients (51.4%) experienced ventilator weaning failure. Specifically, 18 patients (16.2%) failed the SBT, while 93 completed the SBT and were subsequently extubated. Of note, one patient who initially failed the SBT was later extubated by the attending physician during the following shift. Among the 94 extubated patients, 28 (29.8%) required NIV due to respiratory dysfunction, and 19 (20.2%) required reintubation within 72 hours. Some of the reintubated patients initially received NIV before progressing to invasive support.

**Table 1.** Patient characteristics. SAPS, simplified acute physiology score; COPD, chronic obstructive pulmonary, disease; PE, pulmonary edema; SBT, spontaneous breathing trial; NIV, non-invasive ventilation; ICU, intensive care unit.

Variables	Weaning success (n = 54)	Weaning failure (n = 57)	*p*
Males, n (%)	28 (51.9)	28 (49.1)	0.774
Age, years, median (IQR)	65.5 (56.0 - 73.3)	68.0 (59.5 - 74.0)	0.581
SAPS 3, mean ± SD	72.2 ± 14.8	74.6 ± 15.1	0.411
COVID-19, n (%)	1 (1.9)	9 (15.8)	0.017
Heart failure, n (%)	4 (7.4)	8 (14.0)	0.362
COPD, n (%)	10 (18.5)	17 (29.8)	0.165
Reason for intubation, n (%)			0.349
Pneumonia	19 (35.2)	28 (49.1)	
Sepsis without pneumonia	12 (22.2)	12 (21.1)	
Acute cardiogenic PE	2 (3.7)	4 (7.0)	
Altered mental status	10 (18.5)	9 (15.8)	
Postoperative care	6 (11.1)	3 (5.3)	
Shock	3 (5.6)	0	
Other	2 (3.7)	1 (1.8)	
Cumulative fluid balance before SBT, mean ± SD	2545.0 ± 4547.5	4206.5 ± 5511.8	0.087
Duration of MV before SBT, median (IQR)	5.0 (3.0 - 9.0)	5.0 (3.0 - 9.0)	0.645
Prophylactic NIV, n (%)	11 (20.4)	14/40 (35.0)	0.157
ICU mortality, n (%)	6 (11.3)	19 (33.9)	0.005
Hospital mortality, n (%)	14 (26.4)	28 (50.0)	0.011

The distribution of mVExUS scores was as follows: 33 patients (29.7%) were classified as mVExUS 0; 42 (37.8%) as mVExUS 1; 28 (25.2%) as mVExUS 2, and 8 (7.2%) as mVExUS 3. Patient characteristics stratified by VExUS score are detailed in Table 2.

**Table 2.** Patient characteristics stratified by mVExUS score (0–1 vs. 2–3). SAPS, simplified acute physiology score; COPD, chronic obstructive pulmonary, disease; PE, pulmonary edema; SBT, spontaneous breathing trial; NIV, non-invasive ventilation.

Variables	mVExUS 0–1 (n = 75)	mVExUS 2–3 (n = 36)	*p*
Males, n (%)	36 (48.0)	20 (55.6)	0.456
Age, years, median (IQR)	67.0 (57.0 – 74.0)	67.5 (55.5 – 73.0)	0.985
SAPS 3, mean ± SD	74.7 ± 15.4	70.8 ± 13.5	0.189
COVID-19, n (%)	6 (8.0)	4 (11.1)	0.725
Heart failure, n (%)	4 (5.3)	8 (22.2)	0.018
COPD, n (%)	17 (22.7)	10 (27.8)	0.557
Reason for intubation, n (%)			0.041
Pneumonia	30 (40.0)	17 (47.2)	
Sepsis without pneumonia	19 (25.3)	5 (13.9)	
Acute cardiogenic PE	2 (2.7)	4 (11.1)	
Altered mental status	11 (14.7)	8 (22.2)	
Postoperative care	9 (12.0)	0	
Shock	3 (4.0)	0	
Other	1 (1.3)	2 (5.6)	
Cumulative fluid balance before SBT, mean ± SD	3771.8 ± 5326.8	2619.9 ± 4603.9	0.268
Duration of MV before SBT, median (IQR)	5.5 (3.0 - 9.8)	4.0 (2.3 - 7.8)	0.091
Prophylactic NIV, n (%)	17/64 (26.6)	8/30 (26.7)	0.991

### Primary outcome

The incidence of weaning failure was higher among patients with mVExUS scores of 2–3 (63.9%) compared to those with scores of 0–1 (45.3%), although the difference did not reach statistical significance (*p* = 0.067). For individual components of the primary outcome, the mVExUS 2–3 group had an SBT failure rate of 16.7%, NIV use of 40.0%, and extubation failure rate of 23.3%, compared to 16.0%, 25.0%, and 18.8%, respectively, in the mVExUS 0–1 group (*p*-values: 0.929, 0.138, and 0.606, respectively).

In a multivariable analysis adjusted for SAPS 3 score, the duration of MV, cumulative fluid balance prior to SBT, lung POCUS score, and mVExUS scores of 2–3 were independently associated with ventilator weaning failure (Table 3). Model assumptions were adequately met. All tolerance values were above 0.923 and variance inflation factors were below 1.083, indicating low multicolinearity. Interaction terms between each continuous predictor and its natural logarithm were non-significant (SAPS 3, *p* = 0.914; duration of MV, *p* = 0.629; cumulative fluid balance prior to the SBT, *p* = 0.710).

**Table 3.** Univariate and multivariate analysis of risk factor for weaning failure and post-extubation respiratory failure. SAPS, simplified acute physiology score; MV, mechanical ventilation; mVExUS, modified venous excess ultrasound; SBT, spontaneous breathing trial; POCUS, point of care ultrasound.

	Univariate analysis		Multivariate analysis	
Outcome / Variables	Unadjusted OR (95% CI)	*p*	Adjusted OR (95% CI)	*p*
**Primary outcome/ Weaning failure**				
SAPS 3	1.011 (0.985 – 1.037)	0.408		
Duration of MV	1.015 (0.913 – 1.129)	0.777		
mVExUS 2 or 3	2.133 (0.941 – 4.835)	0.069	2.756 (1.093 – 6.944)	0.032
Lung POCUS score ≥ 7.0	3.208 (1.440 – 7.151)	0.004	3.110 (1.286 – 7.520)	0.012
Cumulative fluid balance before SBT	1.000 (1.000 – 1.000)	0.093		
**Secondary outcome/ Post-extubation respiratory failure**				
SAPS 3	1.009 (0.981 – 1.037)	0.538		
Duration of MV	1.041 (0.928 – 1.167)	0.497	1.153 (1.001 – 1.329)	0.048
mVExUS 2 or 3	3.000 (1.191 – 7.558)	0.020	4.454 (1.496 – 13.258)	0.007
Lung POCUS score ≥ 7.0	3.927 (1.602 – 9.625)	0.003	5.123 (1.827 – 14.363)	0.002
Cumulative fluid balance before SBT	1.000 (1.000 – 1.000)	0.132		

### Secondary outcome

Patients with mVExUS scores of 2–3 had a significantly higher incidence of post-extubation respiratory failure compared to those with scores of 0–1 (70.0% vs. 43.8%; *p* = 0.018). This association remained significant in an adjusted analysis, controlling for SAPS 3, duration of MV, cumulative fluid balance, and lung POCUS score (Table 3).

Additionally, we performed an exploratory analysis using a composite outcome that included either weaning failure or post-extubation respiratory failure. mVExUS remained significantly associated with this composite outcome after adjustment (Supplementary Material S1).

### Lung POCUS score

A lung POCUS score ≥7.0 was significantly associated with both primary and secondary outcomes in both unadjusted and adjusted analyses (Table 3). Patients with lung POCUS scores ≥7.0 had a weaning failure rate of 68.8%, compared to 40.7% among those with scores <7.0 (*p* = 0.004). Among patients with lung POCUS scores ≥7.0, 22.9% experienced SBT failure, 50.0% required NIV, and 23.7% had extubation failure, compared to 11.9%, 17.3%, and 19.2%, respectively, in those with scores <7.0 (*p* = 0.128, 0.001, and 0.609).

For the secondary outcome, the incidence of post-extubation respiratory failure was significantly higher in patients with lung POCUS scores ≥7.0 (71.1%) compared to those with scores <7.0 (38.5%; *p* = 0.002).

### Combined mVExUS and lung POCUS scores

**Figure 1.**
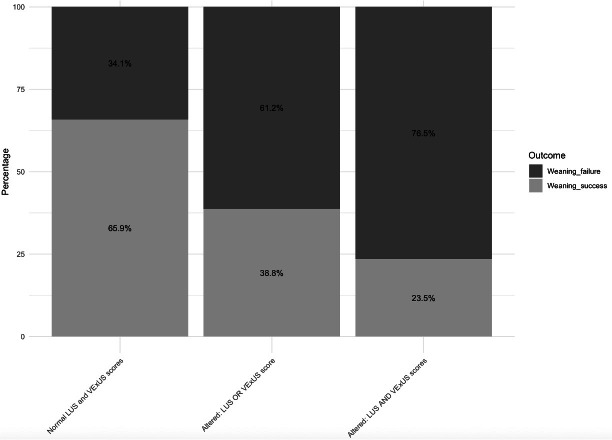
Incidence of weaning failure with combined assessment of mVExUS and lung POCUS scores.

In a post hoc exploratory analysis, the combination of mVExUS and lung POCUS scores was significantly associated with ventilator weaning failure (Figure 1). Patients with both mVExUS scores of 2–3 and lung POCUS scores ≥7.0 had a high incidence of weaning failure (76.5%), with an odds ratio (OR) of 8.3 (95% confidence interval [CI]: 2.114–32.739; *p* = 0.002). In contrast, patients with mVExUS scores of 0–1 and lung POCUS scores <7.0 had a substantially lower incidence of weaning failure (34.1%).

## Discussion

We identified a significant association between mVExUS scores of 2–3 and an increased incidence of both weaning failure and post-extubation respiratory failure. Additionally, our exploratory analysis suggests that combining mVExUS and lung POCUS scores may enhance the evaluation of weaning readiness in critically ill patients.

An inadequate cardiovascular response is a key contributor to weaning failure [3–5]. The transition from MV to spontaneous breathing induces a sudden increase in venous return and left ventricular afterload. This hemodynamic shift can overwhelm patients with subclinical or residual congestion, precipitating WIPO [5,21]. In critically ill populations, occult cardiac dysfunction is common and may compromise compensatory cardiovascular mechanisms [20]. Therefore, identifying patients with clinically relevant systemic congestion remains a priority—albeit a challenging one—in optimizing weaning strategies.

The VExUS score has emerged as a practical and non-invasive bedside tool for assessing systemic venous congestion [11]. It has shown correlations with cardiac filling pressures and associations with the development of acute kidney injury [16,23,24]. Additionally, VExUS has been used to guide decongestion strategies in patients with renal dysfunction [18,25]. Of particular relevance, Longino et al. demonstrated a correlation between VExUS scores and pulmonary capillary wedge pressure, indicating the score's ability to detect elevated left atrial pressure even before overt venous congestion is evident [23]. These findings support our observation that the mVExUS score was independently associated with weaning failure, regardless of lung POCUS findings.

The rationale for combining VExUS and lung POCUS lies in achieving a more comprehensive evaluation of the hemodynamic spectrum of congestion. While VExUS is specifically designed to assess venous congestion, it does not capture interstitial or pulmonary congestion [25]. Conversely, lung POCUS effectively detects pulmonary congestion and has been previously associated with weaning failure [10]. By integrating both tools, clinicians may identify a broader range of congestion phenotypes, potentially improving risk stratification during the weaning process. In our study, we chose to use a lung POCUS score that is well established in the prediction of ventilator weaning failure, rather than limiting the analysis to findings specifically associated with cardiogenic pulmonary edema. This approach allowed us to investigate whether the identification of systemic venous congestion through mVExUS could provide additional prognostic information beyond the pulmonary findings already captured by lung POCUS, even if these are not exclusively attributable to cardiovascular dysfunction.

If validated in future studies, the combined use of VExUS and lung POCUS scores could enhance the early identification of patients at high risk for weaning failure due to cardiovascular dysfunction. This, in turn, could inform tailored management strategies such as judicious use of diuretics, control of hypertension, vasodilator therapy, or the prophylactic application of NIV in the post-extubation period [22]. Similar to the role of B-type natriuretic peptide and echocardiography-guided therapy, the VExUS score may become a useful adjunct in guiding decongestive management during weaning [26,27].

This study had several limitations. First, it was conducted at a single center with a relatively small sample size, which may affect the generalizability of the results. Second, invasive hemodynamic monitoring was not included to confirm the cardiac origin of respiratory failure. Third, a modified version of the VExUS score was applied, which excluded the IRVD component. Although this version is less extensively validated than the original VExUS C score, it has been used in previous studies and has demonstrated good performance in identifying clinically significant venous congestion [16–18]. However, the absence of IRVD may have led to underestimation of congestion in patients with isolated intrarenal abnormalities, while the use of the VExUS A classification—where mild abnormalities are categorized as moderate congestion—may have overestimated severity in others. These classification differences may affect internal validity and limit the comparability of our results with studies using the full VExUS C protocol. Fourth, the cutoff point for the lung POCUS score was derived using Youden's index based on the study dataset, which may overestimate the model's discriminative performance. Although this threshold is consistente with prior literature, such data-driven cutoffs should be interpreted with caution. Fifth, the ventilator weaning failure rate observed in our cohort (51.7%) was relatively high. This may limit the generalizability of our findings to less severely ill populations. However, our definition of weaning failure included SBT failure, reintubation, and the need for NIV within 72 hours, whereas some previous studies used narrower definitions. Moreover, the high severity of illness in our population may have contributed to the elevated failure rate. Finally, the study may be subject to selection bias, as inclusion depended on the availability of a single investigator to perform ultrasound assessment. In addition, despite multivariable adjustment, residual confounding cannot be excluded due to the observational nature of the study. In particular, we did not account for potentially relevant confounders such as detailed comorbidities or treatment strategies, which could influence both venous congestion, lung aeration, and weaning outcomes. This omission should be considered when interpreting our results.

## Conclusions

We observed a significant association between higher mVExUS scores (2–3) and increased incidence of ventilator weaning failure, independent of the lung POCUS score. These findings underscore the importance of evaluating systemic congestion during the weaning process. Furthermore, our exploratory analysis supports the potential utility of combining mVExUS and lung POCUS scores to improve the assessment of weaning readiness. Future research should explore the synergistic application of these tools to optimize patient outcomes during the critical transition from MV.
