# Supplementary material for: Modified Venous Excess Ultrasound (mVExUS) in the Prediction of Ventilator Weaning Failure: a Cohort Study
Source: POCUS J. 2026 Apr 22;11(1):37–44. doi: 10.24908/pocusj.v11i01.19496 (PMC13161785; doi:10.24908/pocusj.v11i01.19496)
Supplement: Supplementary file 1 [file pocusj-11-01-19496-s001.pdf]

Supplementary Material S1. Association between mVExUS and lung POCUS scores and the composite outcome of weaning failure or post-extubation respiratory failure

	Univariate analysis		Multivariate analysis	
Outcome / Variables	Unadjusted OR (95% CI)	*p*	Adjusted OR (95% CI)	*p*
**Composite outcome**				
SAPS 3	1.010 (0.984 – 1.036)	0.452		
Duration of MV	1.038 (0.931 – 1.158)	0.503		
mVExUS 2 or 3	2.769 (1.149 – 6.676)	0.023	3.644 (1.337 – 9.932)	0.011
lung POCUS score ≥7.0	3.987 (1.711 – 9.288)	0.001	4.464 (1.727 – 11.539)	0.002
Cumulative fluid balance before SBT	1.000 (1.000 – 1.000)	0.070		

SAPS, simplified acute physiology score; MV, mechanical ventilation; mVExUS, modified venous excess ultrasound; POCUS, point of care ultrasound; SBT, spontaneous breathing trial
